# *Didymella fabae* Punith.: mating type occurrence, distribution and phenotyping of the anamorph *Ascochyta fabae* Speg. in Tunisia

**DOI:** 10.3389/fpls.2023.1176517

**Published:** 2023-09-04

**Authors:** Noura Omri Ben Youssef, Imen Halila, Ahlem Mbazia, Zayneb Bessaidi, Khawla Missaoui, Mohamed Kharrat, Christophe Le May

**Affiliations:** ^1^ Laboratoire des Grandes Cultures, Institut National de la Recherche Agronomique de Tunisie, Université de Carthage, Ariana, Tunisia; ^2^ INRA, Unité Mixte de Recherche (UMR) 1349 Institut de Génétique, environnement et Protection des Plantes (IGEPP), Le Rheu, France; ^3^ Institut Agro-Rennes-Angers, UP ESP, Rennes, France

**Keywords:** faba bean, ascochyta blight, morphological diversity, conidia, *Didymella fabae*, mating type

## Abstract

Faba bean ascochyta blight, caused by *Ascochyta fabae* Speg. (teleomorph: *Didymella fabae* Punith.), is one of the most devastating diseases of the crop. It can cause yield losses that reach 95% in conducive weather conditions. Surveys were carried out in five regions of Tunisia: Beja, Bizerte, Jendouba, Kef and Tunis-Cap Bon. A total of 513 fungal isolates were collected from 2011 to 2013. A molecular characterization was conducted to identify the mating type of each individual using a mating type specific PCR. Results revealed that the two mating types MAT1-2 and MAT1-1 coexisted in all surveyed regions. An imbalance in favor of MAT1-2 was observed particularly in Bizerte and Jendouba regions (sex ratio was 18:85 and 32:80, respectively). Moreover, morphological and pathogenic characterization of 122 isolates among the collection revealed a significant variability in conidia type (one celled or two celled conidia) frequency, in conidia mean size and in aggressiveness toward Badii faba bean cultivar (incubation period, IP; percentage necrotic leaf area, S; and area under disease progression curve, AUDPC). A principal component analysis (PCA) performed on morphologically studied parameters (frequency of conidia cell number and conidia mean size) identified three groups of isolates based on morphological traits: one celled (1C) and two celled (2C) conidia rates, one celled and two celled conidia length and width (1L, 1W, 2L and 2W, respectively). A second PCA using aggressiveness parameters (IP: Incubation period, S1, S4 and S9: percentage of necrotic leaf area respectively 5, 20 and 45 days after inoculation) identified three distinct pathogenic groups: poorly pathogenic AG1, moderately pathogenic AG2 and highly pathogenic AG3. Morphological and pathogenic groups and mating type data were used to conduct a multiple factorial correspondence analysis (MFCA) which revealed a correlation between the variables studied. Five groups were identified, each associated with a morphological and pathogenic trait and mating type. The most pathogenic group belonged to MAT1-2 suggesting that in locations where MAT1-2 is prevalent the epidemic risk is more important.

## Introduction

Faba bean is one of the most commonly grown legumes in Tunisia. Ascochyta blight caused by *Didymella fabae* Jellis and Punith (anamorph *Ascochyta fabae* Speg.) is a widely spread disease throughout the world particularly in temperate regions (Maurin et al., 1990). It is also one of the most destructive foliar diseases of faba bean fields in Tunisia (Kharrat et al., 2006). The pathogen infects all aerial part of the plant causing necrotic lesions on leaves and pods and enlarged lesions on the stem and the petiole. It leads to yield losses estimated at 10 - 30% which may reach 95% in favorable conditions (Hanounik, 1980; Kharrat et al., 2006).

*D. fabae* (anamoph: *Ascochyta fabae* Speg*.)* was first described in England in 1991 by Jellis and Punithalingam. The genus *Didymella* is part of Didymellaceae Family, Pleosporales Order, Dothideomycetes Class, Pezizomycotina Sub-phylum and Ascomycota Phylum (Nasraoui, 2015). The anamorph, *A. fabae*, Speg. first described in Argentina by Spegazzini in 1899 (Maurin et al., 1990), belongs to the group of Deuteromycetes (imperfect fungi). It is a phialospore species that is part of the subgroup of anamorphic pycnidia fungi (Nasraoui, 2015). *A. fabae* grows on oat agar (OA) medium, in a yellowish colony (Jellis and Punithalingam, 1991). Pycnidia represent fruiting bodies of asexual reproduction that produce hyaline, elliptical, straight or slightly curved conidia with a truncated or rounded base and a rounded apex (Jellis and Punithalingam, 1991). They are predominantly two celled (97%) with a nucleus in each cell (Peever, 2007). Their dimensions are 16-19 x 3.5-4.5 µm (Jellis and Punithalingam, 1991; Omri Ben Youssef et al., 2012). Several studies have reported significant variability of morphological characters within populations of *A. fabae* (Kharbanda and Bernier, 1980; Maurin and Tivoli, 1992; Kohpina et al., 1999; Kharrat et al., 2000). Various parameters relating to *A. fabae* development were considered, including mycelium aspect (aerial or diffuse…), pycnidia pigmentation (brown, black, orange) and colony margin (regular or irregular). The characterization of 30 isolates of *A. fabae* from Tunisia, France and Algeria, carried out by Kharrat et al. (2000), confirmed this morphological variability. However, the diversity of these criteria appeared to be not linked to the geographic origin of the strains especially since Maurin (1989) observed significant variability even among conidia originating from the same pycnidia.

The pathogenicity variation of *A. fabae* has been reported in numerous studies (Hanounik and Robertson, 1989; Maurin, 1989; Rashid et al., 1991; Onfroy et al., 1999; Kharrat et al., 2000; Omri Ben Youssef et al., 2016; Blake et al., 2022). All these studies showed significant different interactions of faba bean lines when inoculated with different isolates suggesting significant pathogenic variability of the fungus and the presence of physiological specialization (Tivoli et al., 2006). However, physiological races in *A. fabae* remain a subject of controversy as some authors support the hypothesis of race presence (Hanounik and Robertson, 1989; Rashid et al., 1991), while others agree on the presence of a certain specialization without being able to identify physiological races (Maurin, 1989; Beed et al., 1994; Kharrat et al., 2000). In fact, *Vicia faba* resistance to *A. fabae* is polygenic and involves simultaneously major genes and other minor genes, making the distinction between the host resistance reaction and sensitivity difficult (Kharrat, 1999).

*Ascochyta fabae* is a haploid heterothallic pathogen that can maintain itself during the off-season on different sources such as seeds and plant debris (Kaiser, 1997; Tivoli and Banniza, 2007). It is maintained on plant debris under both anamorph and teleomorph forms for at least one season (Gossen and Morrall, 1986; Pedersen et al., 1993; Rubiales and Trapero-Casas, 2002; Omri Ben Youssef et al., 2012). In heterothallic species, the mating-type locus contains one of two dissimilar forms of sequences called idiomorphs since the alternative alleles of mating-type locus are dissimilar, but are located at the same chromosomal location within the genome (Metzenberg and Glass, 1990). Conventionally, mating type idiomorphs of complementary isolates are called MAT1-1 and MAT1-2 (Turgeon and Yoder, 2000). In Dothideomycetes and particularly in *Didymella* genus, the idiomorph MAT1-1 corresponds to a unique regulatory gene MAT1-1-1 characterized by the presence of an ORF (Open Reading Frame) region encoding a protein with a motif called the α domain, initially discovered in the MATα1 transcription factor of *Saccharomyces cerevisiae* (Barve et al., 2003; Chérif et al., 2006). The idiomorph MAT-1-2 corresponds to the MAT-1-2-1 gene, characterized by the presence of an ORF zone encoding a regulatory protein with a high mobility domain (HMG) (Turgeon et al., 1993; Coppin et al., 1997; Turgeon, 1998; Turgeon and Yoder, 2000; Barve et al., 2003; Chérif et al., 2006; Debuchy and Turgeon, 2006). Idiomorphs are flanked by DNA sequences, common to both sex types (Kronstad and Staben, 1997), with unknown molecular function but not essential for reproduction (Debuchy and Turgeon, 2006).The development of specific primers to each idiomorphic sequence at the MAT locus of *A. fabae* (Chérif et al., 2006) made it possible to address questions such as the spatial and temporal distribution of these mating types, their respective prevalence within populations and finally the impact of this distribution on the diversity and genetic structure of pathogen populations (Milgroom, 1996; Barve et al., 2003; Chérif et al., 2006). The first studies dealing with *A. fabae* mating type distribution and involvement in genetic structure were those of Ozkilinc et al. (2015) and Omri Ben Youssef et al. (2019) who studied the role of mating types in the population diversity of Syrian and Tunisian collections of *A. fabae*. Omri Ben Youssef et al. (2019) showed that among their 240 Tunisian isolates MAT1-2 was more common in Tunisia than MAT1-1, and Tunisian populations had a skewed distribution (1:2 ratio) for MAT1-1:MAT1-2. Of the four locations studied, two (Beja and Tunis) gave a 1:1 distribution while for the other two locations, MAT1-2 was more common than MAT1-1. However, Ozkilinc et al. (2015) showed that 1:1 ratio could not be rejected for two populations of *A. fabae* from Syria although a random mating hypothesis was rejected in these populations. In contrast, a study conducted on 311 isolates of *A. fabae* collected between 1991 and 2018 in South Australia by Blake et al. (2022) showed an equal ratio of MAT1–1 and MAT1–2. Moreover, this study came to a conclusion that there is no relationship between isolate aggressiveness and mating type.

The presence of two mating types can induce teleomorph development of *D. fabae* and by genetic recombination, new virulent strains can emerge. However, the presence of two mating types in the same region or field does not lead automatically to sexual recombination as revealed by Ozkilinc et al. (2015) and Omri Ben Youssef et al. (2019). The trend toward clonal reproduction in the same region or field could be due to a temporal and/or spatial lag in the development of the two mating types. The time lag could originate from different optima of the climatic parameters for the development of the two sexual types, particularly temperature and humidity, two key parameters for reproduction (Maurin et al., 1990). Variability in optimal temperature was reported for *A. fabae* (Kharrat, 2003) and other *Ascochyta* species (Le May et al., 2012). However, this variability has so far not been linked to mating type. A spatial lag would work on a lower scale than the field (plant and/or its different organs). Indeed, mating types may develop in the same field but on different plants or different organs (stem or leaf) making their recombination difficult as suggested by Omri Ben Youssef et al. (2019).

The present study aimed to: (i) assess the frequency and the distribution of both *A. fabae* mating types among different locations in a larger Tunisian population to confirm the findings of Omri Ben Youssef et al. (2019); (ii) define the aggressiveness level and the morphological features of *A. fabae* isolates belonging to each mating type, and (iii) define if the membership to a mating type is associated with particular phenotypic traits or aggressiveness level.

## Material and methods

### Fungal isolates

**Figure 1:**
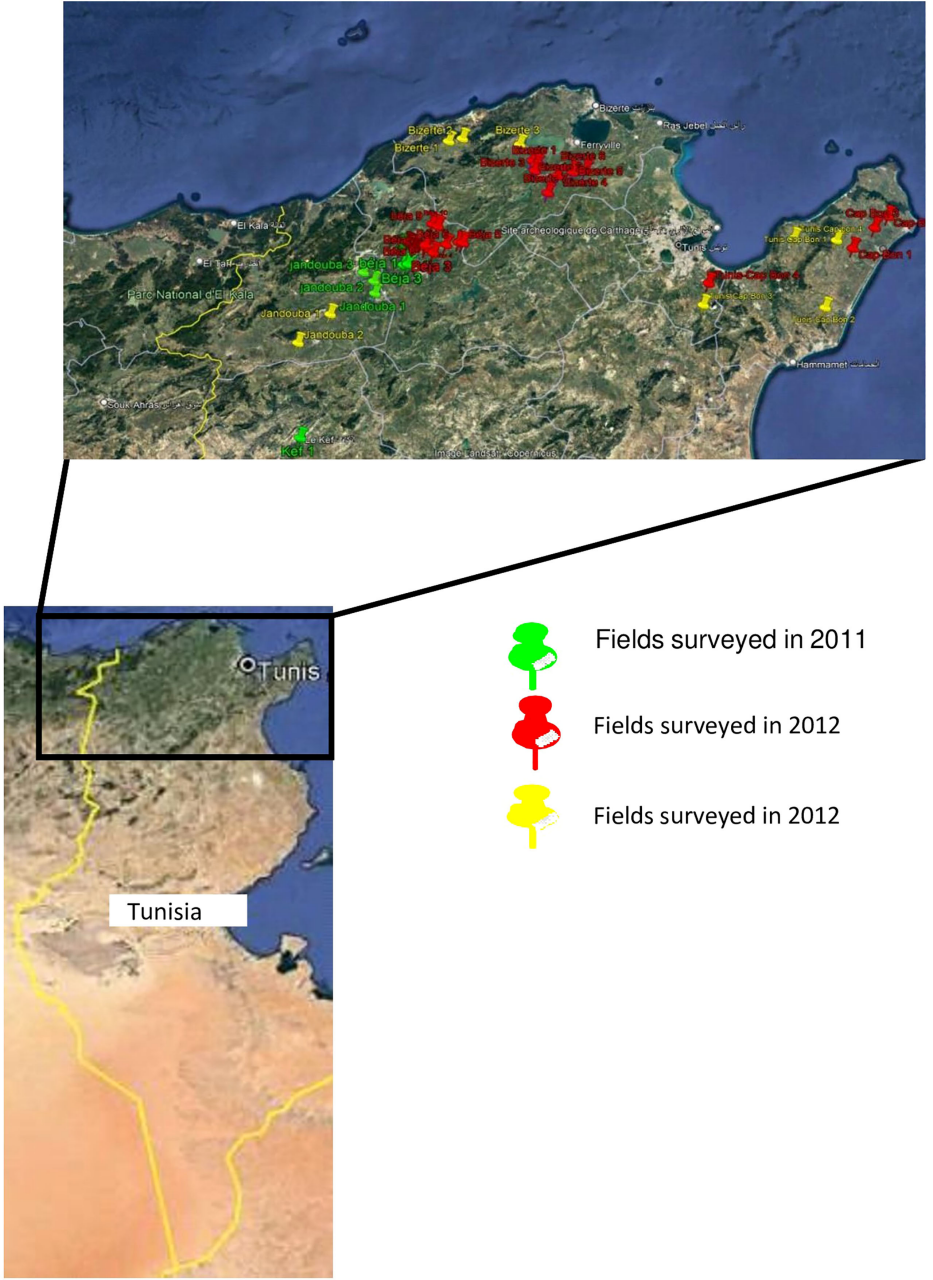
Fields surveyed to collect *Ascochyta fabae* in 2011, 2012 and 2013.

A total of 513 A*. fabae* isolates were used in this study. All the isolates were collected between 2011 and 2013 in several fields from five different geographical region locations in Tunisia, namely Beja (198 isolates: B1-B198), Bizerte (103 isolates: Bz1-Bz103), Jendouba (112 isolates: J1-J112), Kef (45 isolates: K1-K45) and Tunis-Cap Bon (55 isolates: TC1-TC55) (**Table 1**, **Figure 1**). All isolates were sampled from infected leaves. Faba bean tissue with lesions were collected randomly at intervals of 3 to 5 km in each region without considering the cultivar. Lesions were cultured on V8 medium in Petri dishes. Resulting conidia were spread onto malt agar and single germinating conidia were transferred to potato dextrose agar (PDA) and incubated at 20°C with a 12h photoperiod under cool white fluorescent lamps as described in Omri Ben Youssef et al. (2019).

**Table 1:** Origin of *Ascochyta fabae* isolates used in the study.

Region	Sampling year	Number of fields	Number of isolates collected
**Beja**	2011	5	34
	2012	10	164
	**Total**	15	198
**Jendouba**	2011	3	27
	2012	5	63
	2013	2	19
	**Total**	10	112
**Bizerte**	2012	7	80
	2013	3	24
	**Total**	10	103
**Kef**	2011	1	45
	**Total**	1	45
**Tunis-Cap Bon**	2012	4	27
	2013	4	28
	**Total**	8	55

Each single spore isolate was then grown in 75ml of peptone liquid (LP) medium supplemented with streptomycin (1.5g) and penicillin (0.75g). Each culture was raised from four pieces (approximately 1cm² each) cut from the margin of an actively growing culture on PDA. Inoculated vials were incubated, under agitation, for 14 days at 20°C with a 12h photoperiod under cool white fluorescent lamps. Mycelia were harvested by vacuum filtration through two layers of sterilized Miracloth (Calbiochem CN Biosciences, Inc., La Jolla, CA), rinsed twice in sterile water and stored at -80°C until lyophilized. DNA was extracted from 1g fungal mycelium and isolated using Nucleospin Plant II Kit (Macherey-Nagel, France) according to manufacturer’s instructions.

### Mating type determination and distribution

Mating type of all the 513 A*. fabae* isolates was determined using the multiplex MAT-specific

PCR assay (Chérif et al., 2006). Primer combinations AL2p2SeqF4 (5’GCAACATCCTAGCATGATG3’) specific to MAT1-1, AL1p1SeqF5 (5’CTGTCTCACCCAAGGCAAAC3’) specific to MAT1-2 and ACom1A1AvAfAp

(5’CACATCACCCCACAAGTCAG3’), specific to an aligning flanking 3’ region of *A. lentis, A. viciae-villosae, A. fabae* and *A. pisi* were used. Single PCR was carried out in 25μl containing 10 ng of genomic DNA, 1X PCR buffer (containing 1.5 mM MgCl_2_), 0.2 mMdNTPs, 1unit Taq DNA polymerase (Promega, USA) and 0.2 µM each of the primers. Amplification was performed in Bio-Rad thermal cycler (Bio-Rad Laboratories, USA) and cycling conditions consisted of an initial denaturation at 95°C for 3 min followed by 35 cycles of 94°C for 20 sec, 58°C for 20 sec, 72°C for 40 sec, and a final extension at 72°C for 10 min. DNA amplicons were separated in 1.5% ethidium bromide-stained agarose gels and visualized under UV light on a ChemiDOCTM XRS documentation system (Bio-Rad, USA). Amplicon size was estimated using a DNA ladder (Hyperladder II, USA). Collected data on mating type were then submitted to Chi-Square test in PROC FREQ of SAS 9.2.

### Isolate phenotyping

122 isolates were sampled randomly among the 513 isolates for phenotyping considering regions and mating types (11 to 13 isolates were selected for each region and each mating type). Shapiro Wilk test, Fischer test and Student t-test were respectively used to assess sample normality to compare variances and independence of subsamples (from each region) for each scored parameter for phenotyping. All these tests gave no significant difference between regions (P>0.05) allowing regional data to be combined in subsequent analyses.

#### Morphological characterization

Morphological phenotyping consisted of evaluating size, shape and conidial septation of the isolates under compound microscope (objective x40). Two slides were prepared from each isolate. Septum type (one celled, 1C and two celled, 2C) was evaluated on two microscope fields and conidial dimensions (one celled and two celled conidia length and width, 1L, 1W, 2L and 2W, respectively) were evaluated on 15 conidia of each preparation, and data transformed to percentage in the two preparation fields. To test individual variation the data of each parameter were subjected to an analysis according to a completely random design using PROCGLM procedure of SAS 9.2 software.

To determine if conidia-associated traits can separate and classify isolates into different groups, a Principal Component Analysis (PCA) was performed considering the mean septum type (1C and 2C) and mean dimensions of each type of conidia (1L, 1W, 2L and 2W). The analysis was performed using the PROC PRINCOMP procedure of the SAS 9.2 software.

#### Aggressiveness characterization

In a total of 366 pots containing potting soil, 2 seeds of Badii faba bean cultivar (released by Field Crop Laboratory of National Agronomic Research Institute of Tunisia in 2006 and known to be sensitive to ascochyta blight) were sown per pot and grown with irrigation under controlled conditions until three leaf stage. To evaluate the aggressiveness level of the 122 selected *A. fabae* isolates, at the three-leaf stage, each of three pots (one pot is considered an experimental unit repeated 3 times) was inoculated with 6 ml of a spore suspension of one of the 122 isolates adjusted to a concentration of 10^5^ spores per ml. The pots were then maintained under 20°C and 16h of photoperiod with misting thrice a day to maintain favorable humidity. For each isolate, the incubation period (IP) was evaluated by measuring the number of days required for the onset of the first symptoms. The disease was also evaluated by visually estimating the percentage of necrotic leaf area (S) on the three first inoculated leaves at a regular interval of five days up to 45 days after inoculation. S1 to S9 were recorded on 5, 10, 15, 20, 25, 30, 35, 40 and 45 days after inoculation, respectively. Each set of data was then submitted to an analysis according to a randomized complete block design to test individual variation.

In order to check if aggressiveness parameters can separate the isolates into distinct groups, a PCA was conducted considering the mean incubation period (IP) and the percentage of necrotic leaf area for S1, S4, and S9. The analysis was performed, using the PROC PRINCOMP procedure of the SAS 9.2 software.

### Statistical analysis of aggressiveness and morphological traits with mating type

In order to identify a link between mating type and phenotypic traits (morphology and aggressiveness) of *A. fabae* individuals, an MFCA (multiple factorial correspondence analysis) was conducted in SAS 9.2, through a multidimensional contingency table (Burt matrix) of all two-way cross-tabulations across all variables. MFCA decomposed the Burt matrix to find the pair wise associations which account for the greatest inertia proportion and displayed them on a reduced number of dimensions. It was carried out considering (i) three morphological groups (MG) obtained from the PCA performed on mean septum type and mean dimensions of each type of conidia, (ii) three aggressiveness groups (AG) obtained from the PCA conducted on the mean incubation period and the percentage of necrotic leaf area, and (iii) two mating types.

## Results

### Mating type occurrence and distribution within different Tunisian locations

**Figure 2:**
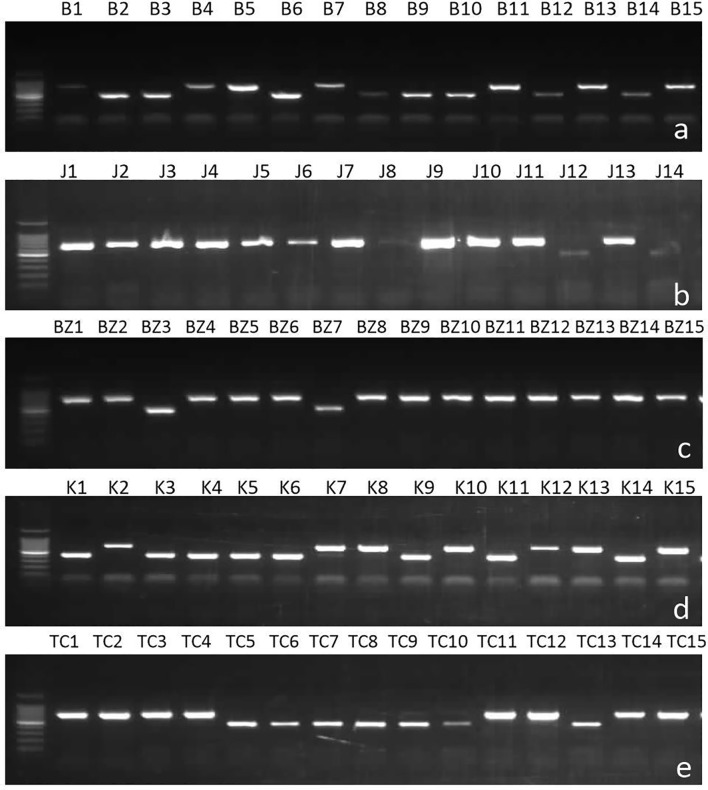
Samples of gels showing result of multiplex PCR analysis of *Ascochyta fabae* mating types in Tunisia with primers AL2p2SeqF4 specific to MAT1-1, AL1p1SeqF5 specific to MAT1-2 and ACom1A1AvAfAp specific to the flanking region. Bands of size 700 bp correspond to MAT 1-2 and bands of 450 bp correspond to MAT 1-1. **(A)** Beja, **(B)** Jendouba, **(C)** Bizerte, **(D)** Kef and **(E)** Tunis-Cap Bon.

Mating type of isolates was determined in PCR analysis using specific mating type primers which amplified one band of 700bp for Mat1-1or 450bp for Mat1-2 (**Figure 2**). Results showed that among the 513 Tunisian isolates, MAT1-2 was more common in the combined Tunisian collection than MAT1-1 (**Table 2**) with the Chi square analysis rejecting random mating (χ^2^ = 39.86, P-value <0.0001). However, after combining the populations of all years for regions where we sampled more than one year (having tested the two/three years independently and identifying no differences) results were different from one region to another.

**Table 2:** Mating type ratios and χ^2^test for random mating within *Ascochyta fabae* populations from Tunisia.

Region	N^a^	Ratio^b^	χ^2c^	*P^d^*
Beja	198	94:104	0.50	0.47
Jendouba	112	32:80	20.57	<0.0001
Bizerte	103	18:85	43.58	<0.0001
Le Kef	45	16:29	3.75	0.05
Tunis-Cap Bon	55	25:30	0.45	0.50
Total	513	185:328	39.86	<0.0001

^a^Number of isolates analyzed.

^b^sex ratio: Mating type 1 (MAT1-1): mating type 2 (MAT1-2).

^c^χ^2^-value for the test of 1:1 ratio.

^d^Probability of greater χ^2^–value under the null hypothesis of 1.

Random mating was rejected using the χ^2^ratio test in Bizerte and Jendouba (χ^2^= 43.58, P- value <0.0001 and χ^2^= 20.57, P-value <0.0001 respectively) but was not rejected in the remaining three regions, Beja (χ^2^= 0.50, P-value = 0.47), Kef (χ^2^= 3.75, P-value = 0.0526) and Tunis-Cap Bon (χ^2^= 0.45, P-value = 0.50) (**Table 2**). For the 103 isolates from Bizerte and the 112 isolates from Jendouba, the two mating type ratios were 18:85 and 32:80, respectively, with greater numbers of MAT1-2 in both these regions.

### Isolate phenotyping

#### Morphological characterization

**Figure 3:**
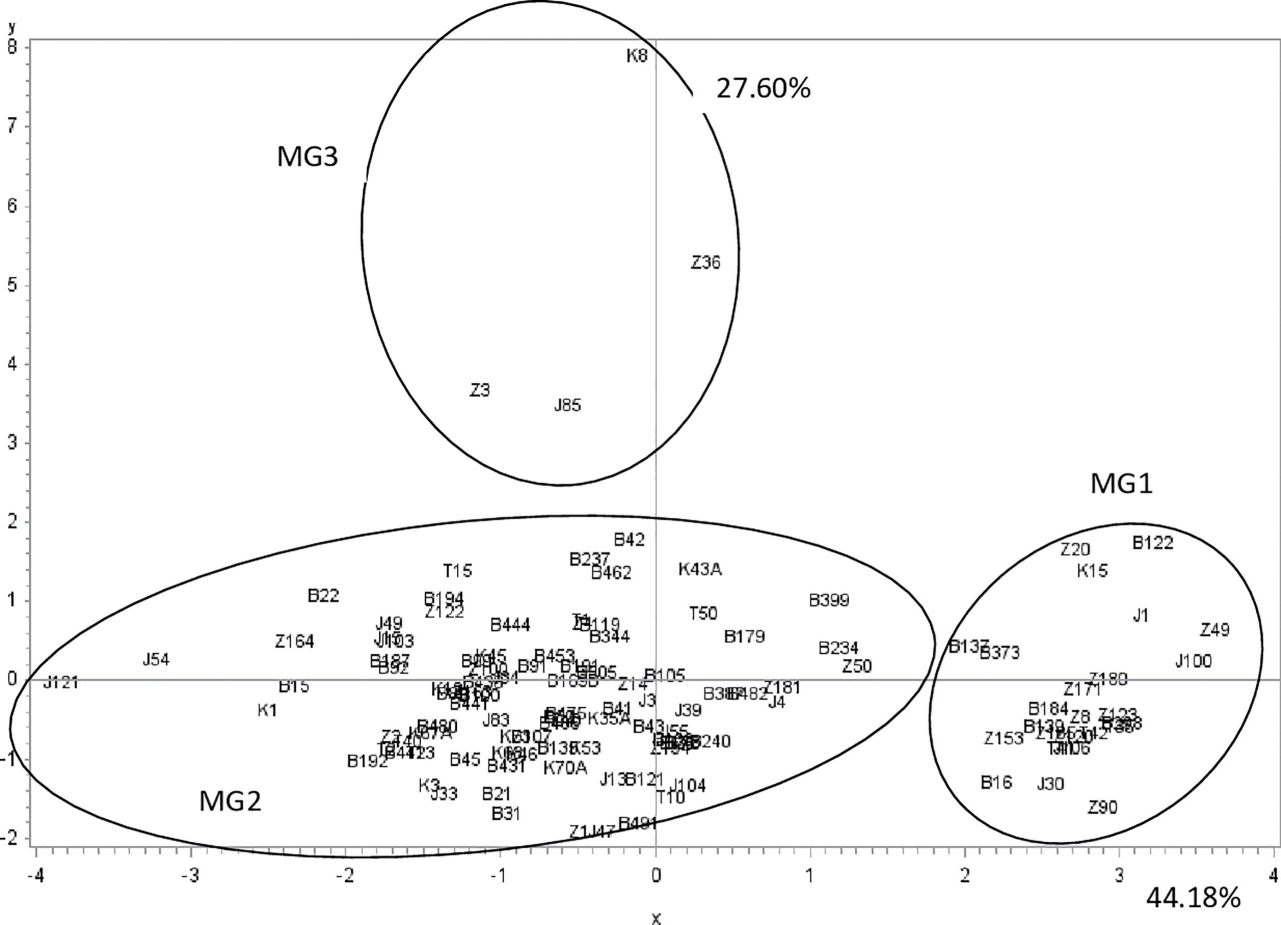
*Ascochyta fabae* isolate position on the two first most representative components (x and y) representing respectively 44.2 and 27.6 percent of variability in morphological parameters (one celled, two celled rate conidia, length and width of one celled and two celled conidia). Z, Bizerte; B, Beja; J, Jendouba; K, Kef and T, Tunis-Cap Bon.

Analysis of variance showed a significant effect (P-value < 0.0001) of the isolate on the different parameters (1C and 2C conidia rates, and conidia dimension: 1L, 1W, 2L and 2W). A PCA was performed on these data to illustrate correlation between these variables and to classify *A. fabae* isolates into morphological groups (MG). Results showed that the first two dimensions explained more than 71% of variation. Component 1 accounted for 44% of data variance and component 2 accounted for 27.60% of data variance (**Figure 3**). The first component was positively correlated with 2C, 2L, 2W, and 1W. It was negatively correlated with 1C and 1L. The second component was positively correlated with 1C, 1W and 2W. It was negatively correlated with 2C, 1L, and 2L (**Table 3**). All morphological parameters were correlated with each other (**Table 4**).

**Table 3:** Pearson Correlation Coefficients of morphological parameters of *Ascochyta fabae* isolates with the first two components (Number of isolates=122, one-celled conidia rate, 1C; two-celled conidia rate, 2C; one-celled conidia length, 1L, one-celled conidia width, 1W; two-celled conidia length, 2L and two celled conidia width, 2W).

Parameter	Prin1	Prin2
**1C**	-0.88	0.39
**2C**	0.88	-0.38
**1L**	-0.83	-0.10
**1W**	0.23	0.90
**2L**	0.18	-0.08
**2W**	0.55	0.73

**Table 4:** PCA Correlation Matrix of morphological parameters of *Ascochyta fabae* isolates (Number of isolates=122, one-celled conidia rate, 1C; two-celled conidia rate, 2C; one-celled conidia length, 1L, one-celled conidia width, 1W; two-celled conidia length, 2L and two celled conidia width, 2W).

	1C	2C	1L	1W	2L	2W
**1C**	1.0000	-.9539	0.5867	0.1021	-.1706	-.2068
**2C**		1.0000	-.6207	-.0891	0.1026	0.2043
**1L**			1.0000	-.2313	-.0256	-.4435
**1W**				1.0000	-.0234	0.6692
**2L**					1.0000	0.0920
**2W**						1.0000

These two components distinguished three groups of isolates (**Figures 3**). The first group (MG1) was composed of isolates with a high frequency of two-celled, large and long conidia (**Table 3**, **Figure 3**). The second group (MG2) was composed of isolates with one and two celled isolates with medium dimensions (**Table 3**, **Figure 3**). The third group (MG3) was composed of isolates with a high frequency of large one celled conidia (**Table 3**, **Figure 3**).

#### Aggressiveness characterization

**Figure 4:**
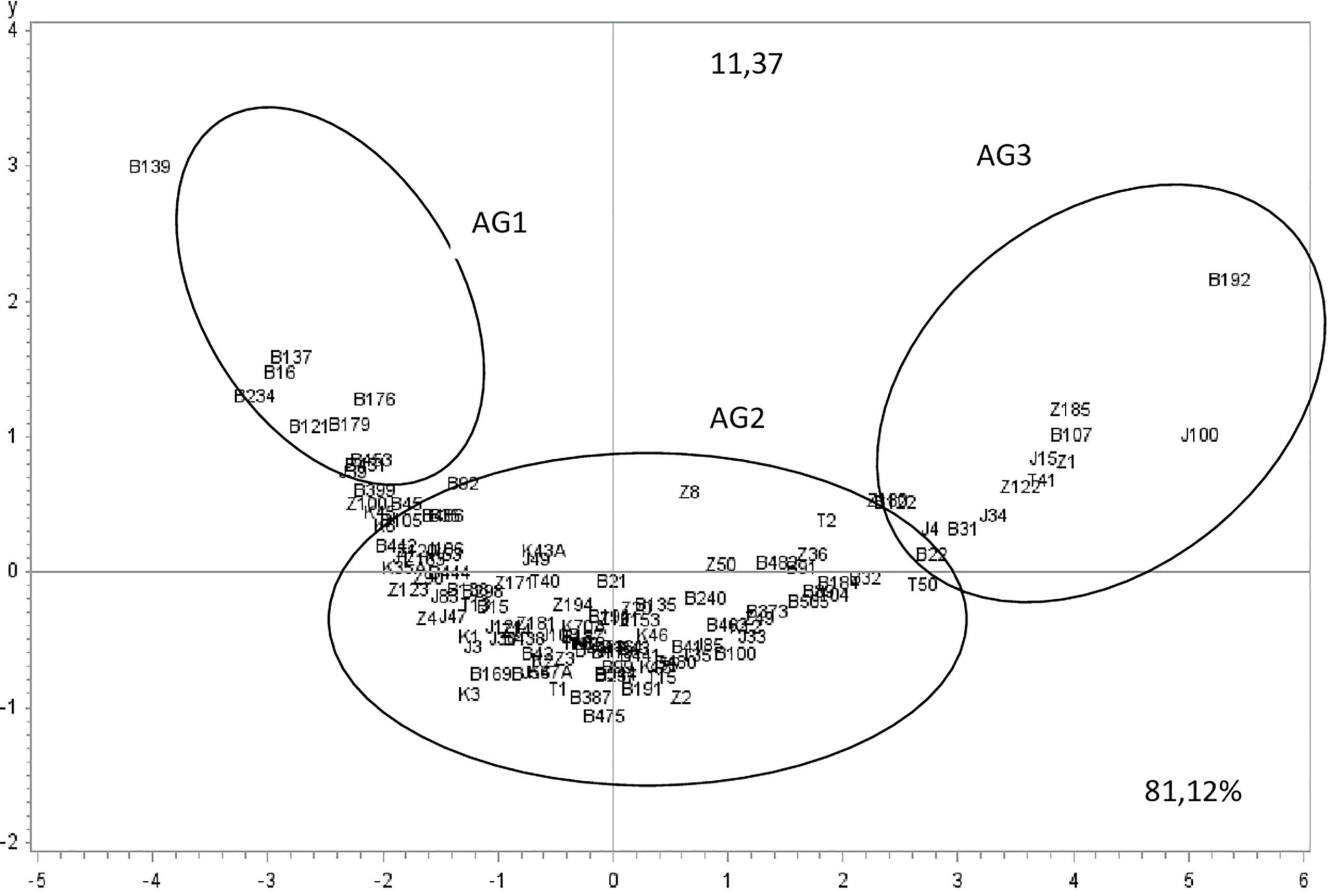
*Ascochyta fabae* isolate position on the two first most representative components (x and y) representing respectively 81.1 and 11.3% of variability in aggressiveness parameters (Incubation period: IP, the percentage of necrotic leaf area: 5,20 and 45 days after inoculation respectively S1, S4, and S9). Z, Bizerte; B, Beja; J, Jendouba; K, Kef and T, Tunis-Cap Bon.

Aggressiveness was significantly different between the tested isolates (P-value <0.0001) for all the parameters assessed (IP, S1, S4, and S9). PCA analysis showed that the two first components explained more than 92% of variation. Component 1 accounted for 81.12% of data variance and component 2 accounted for 11.37% of data variance (**Figure 4**). The first component was positively correlated with S1, S4 and S9, and was negatively correlated with IP. The second component was positively correlated with IP, S1, and S4. It was negatively correlated with S9 (**Table 5**). All scored aggressiveness parameters were correlated with each other (**Table 6**).

**Table 5:** Pearson correlation coefficients of aggressiveness parameters of *Ascochyta fabae* isolates with the first two components (PC1 and PC2) revealed by PCA conducted on 122 isolates and considering incubation length period in days, IP: and scoring of the percentage of necrotic leaf area: 5, 20 and 45 days after inoculation of faba bean cultivar Badii, respectively S1, S4 and S9.

	PC1	PC2
**IP**	-0.812	0.566
**S1**	0.883	0.302
**S4**	0.953	0.207
**S9**	0.946	-0.004

**Table 6:** PCA Correlation Matrix of aggressiveness parameters of *Ascochyta fabae* isolatesrevealed by PCA conducted on122 isolates and considering incubation length period in days, **IP**: and scoring of the percentage of necrotic leaf area: 5, 20 and 45 days after inoculation of faba bean cultivar Badii, respectively **S1, S4** and **S9**.

	IP	S1	S4	S9
**IP**	1.0000	-.5929	-.6484	-.7279
**S1**		1.0000	0.9577	0.7379
**S4**			1.0000	0.8415
**S9**				1.0000

**Figure 5:**
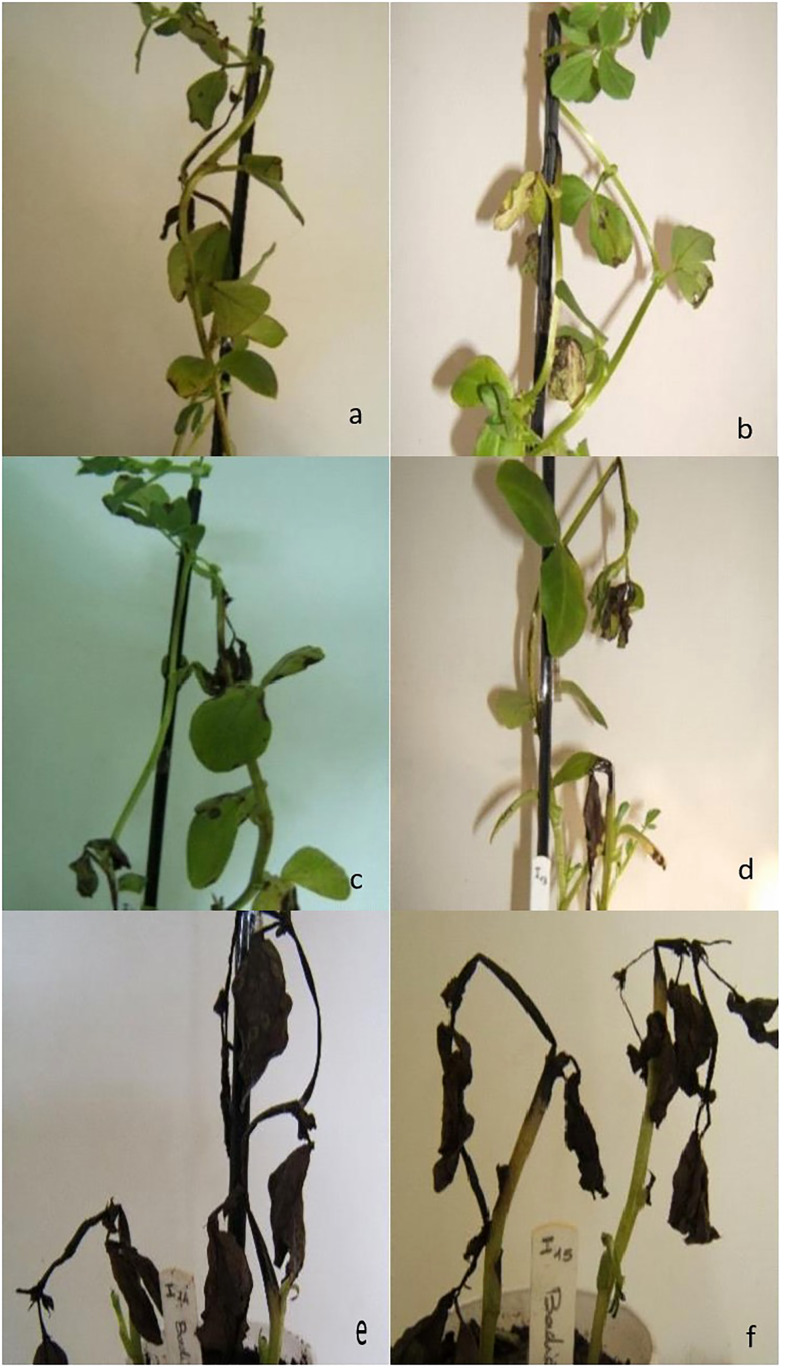
Reaction of faba bean Badii cultivar to non-aggressive **(A, B)**, moderately aggressive **(C, D)** and highly aggressive **(E, F)** *Ascochyta fabae* isolates.

The two components distinguished three isolate groups (**Figures 4**, **5**). The first group: aggressiveness group1 (AG1), was composed of 12 isolates with long incubation period and low disease scores (S1, S4 and S9) and mostly originated from Beja region. The second group: aggressiveness group2 (AG2), was composed of 95 isolates with medium values of all parameters (IP and S1, S4 and S9) and categorized as moderately pathogenic. Isolates belonging to this group originated from all regions. The third group: aggressiveness group3, (AG3) was composed of 15 isolates having high disease scores (S1, S4 and S9) and low IP. This group was categorized as the most pathogenic. Isolates belonging to this group mostly originated from Beja, Bizerte and Jendouba regions.

### Statistical analysis of aggressiveness and morphological traits with mating type

**Figure 6:**
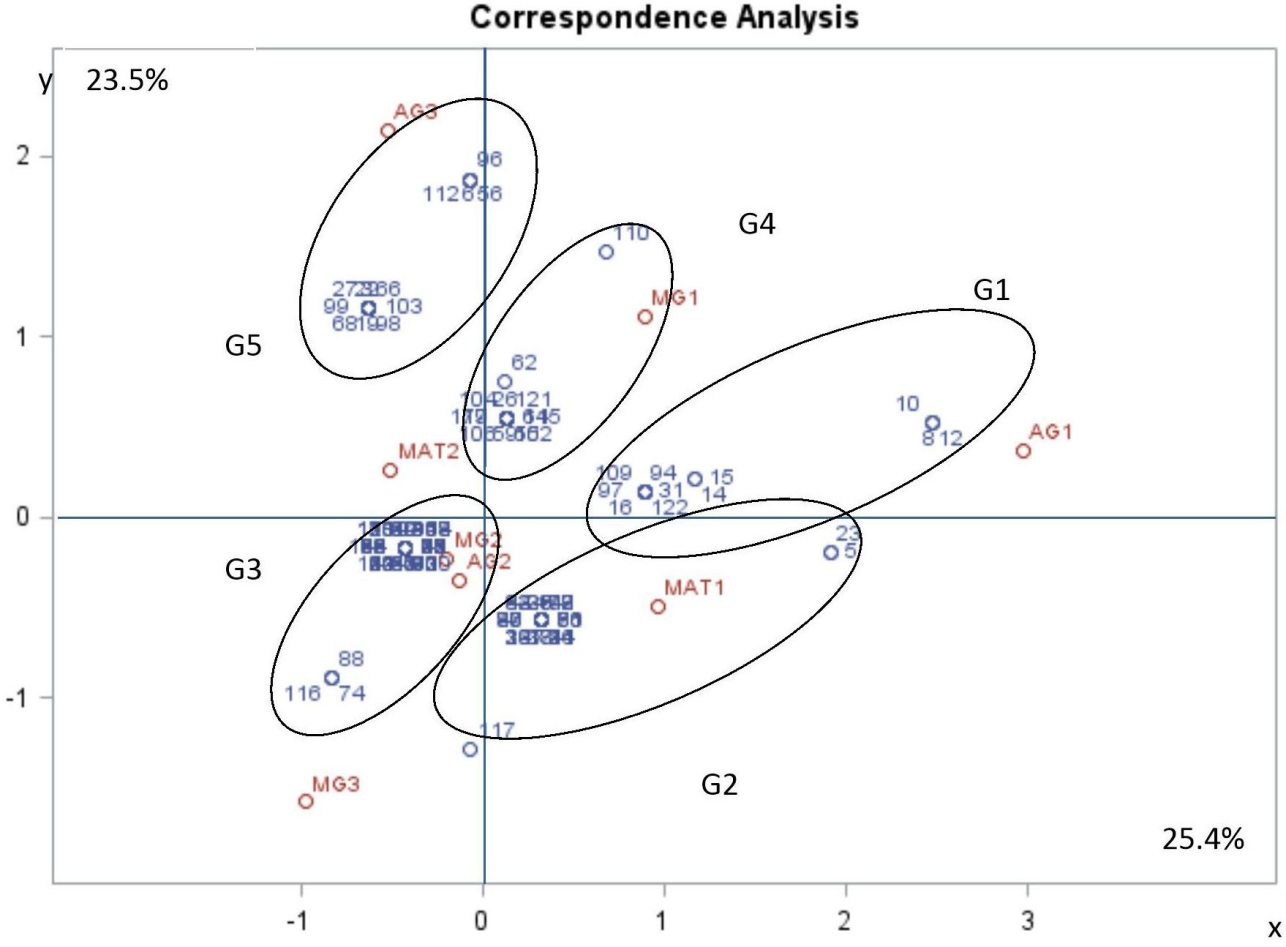
Multiple factorial correspondence analysis (MFCA) performed on *Ascochyta fabae* isolates regarding their mating type (MAT1-1 and MAT1-2), their aggressiveness group (AG1, AG2 and AG3) and their morphological group (MG1, MG2 and MG3). Aggressiveness group (AG1: poorly pathogenic, AG2: moderately pathogenic and AG3: highly pathogenic), Mating type (MAT1: mating type 1 and MAT2: mating type 2) and morphological group (MG1, MG2 and MG3). The two first most representative components (x and y) represent respectively 25.4 and 23.5 percent of variability. Variable correlation with two components (in red) and Isolate position on two components (in blue).

The purpose in using MFCA was to establish whether there was a correlation between morphological traits, mating type and aggressiveness groups. Based on the values of inertia and Chi square, the first dimensions were considered the most representative and accounted for almost 49% of variation, with dimension 1 and 2 representing respectively more than 25% and more than 23% of the variation. Dimension 1 is positively correlated with MAT1-1, MG1 and AG1, and negatively correlated with MAT1-2, MG2, MG3, AG2 and AG3. Dimension 2 is positively correlated with MAT1-2, MG1, AG1 and AG3. It is negatively correlated with MAT1-1, MG2, MG3 and AG2 (**Table 7**, **Figure 6**). The two components distinguished five groups of isolates (G1 to G5) according to their pathogenicity, morphological traits and mating type (**Figure 6**, **Table 8**). G1 was composed of isolates belonging to MAT1-1, to the moderately to poorly pathogenic group1 (AG2 and AG1) and mostly MG1 morphological traits of two-celled, large and long conidia. G2 was composed of isolates belonging to MAT1-1, to the moderately pathogenic group (AG2) and mostly MG2 morphological traits of one and two-celled isolates with medium dimensions and isolates with long one-celled conidia. G3 was composed of isolates belonging to MAT1-2, to the moderately pathogenic group (AG2) and mostly MG2 and MG3 morphological traits. G4 was composed of isolates belonging to MAT1-2, to the moderately pathogenic group (AG2) and MG1 morphological traits. G5 was composed exclusively of the most pathogenic isolates toward cv. Badii, belong to MAT1-2 group and mostly MG2 morphological traits (isolates with one and two-celled isolates with medium dimensions).

**Table 7:** Coordinates of the different groups of *Ascochyta fabae* isolates with the first two components revealed by FMCA conducted on 122 isolates and considering mating type 1, MAT1-1; mating type 2, MAT1-2; Morphological group 1,2 and 3 respectively, MG1, MG2, MG3 and, Aggressiveness group 1, 2 and 3 respectively, AG1, AG2, AG3.

	PC1	PC2
**MAT1-1**	0.966	-0.495
**MAT1-2**	-0.507	0.259
**MG1**	0.897	1.111
**MG2**	-0.199	-0.230
**MG3**	-0.978	-1.575
**AG1**	2.978	0.370
**AG2**	-0.129	-0.347
**AG3**	-0.527	2.143

**Table 8:** Morphological traits, aggressiveness group and mating type of isolate groups defined by projection on the first two components (MAT1-1: mating type1, MAT1-2: mating type 2, MG1, MG2, MG3: Morphological group 1,2 and 3 respectively, AG1, AG2, AG3: Aggressiveness group 1, 2 and 3, respectively.

Group	Mating type	Morphological group	Aggressiveness group
**G1**	MAT1-1	AG1 and AG2	MG1
**G2**	MAT1-1	AG2	MG2
**G3**	MAT1-2	AG2	MG2 and MG3
**G4**	MAT1-2	AG2	MG1
**G5**	MAT1-2	AG3	MG1 and MG2

## Discussion

The main objectives of this study were to assess frequency and distribution of both *A. fabae* mating types among different locations in Tunisia, to define aggressiveness level and morphological features of *A. fabae* isolates belonging to each mating type, and to check if the membership to a mating type would be associated to particular phenotypic traits or aggressiveness level. The *D. fabae* teleomorph has been reported in many countries: Canada (Kharbanda and Bernier, 1980), Poland (Filipowicz, 1983), United Kingdom (Jellis and Punithalingam, 1991), Australia (Kaiser, 1997), Syria (Bayaa and Kabbabeh, 2000), Spain (Rubiales and Trapero-Casas, 2002) and more recently in Tunisia (Omri Ben Youssef et al., 2012). Since the discovery of the teleomorph form in Tunisia, several questions arose regarding the spatial distribution of these mating types and their respective prevalence within populations (Ozkilinc et al., 2015; Omri Ben Youssef et al., 2019; Blake et al., 2022). Our study showed that MAT1-2 was more common than MAT1-1 in three Tunisian locations (Bizerte, Jendouba and Kef), and for two locations (Beja and Tunis-Cap Bon) sexual reproduction was regularly observed. Indeed, Beja and Tunis-Cap Bon were known to be hot spots of *A*. *fabae* and are the main faba bean growing regions. The minor type of faba bean (*Vicia faba* minor) are most commonly grown in Beja and Tunis-Cap Bon and possibly the majority of disease samples in those regions came from the minor type crop. In contrast, both minor and major type (*Vicia faba* major) are grown in Jendouba and Bizerte so here the disease samplings were likely to be collected equally from both types. One hypothesis is that mating type may be linked to host type (minor or major) since pathogens can be subjected to host selection as mentioned by McDonald and Linde (2002) and De Meeûs et al. (2007). Host cultivar or faba bean type was not considered in the data analyses in this study but it could be an area of further study. These results confirmed an earlier study conducted by Omri Ben Youssef et al. (2019) on a smaller population which concluded that sexual recombination does not play an important role in fungus diversity through these regions. The same conclusion was reported by Ozkilinc et al. (2015) in their study on Syrian populations. In contrast, Blake et al. (2022) reported an equal ratio of MAT1–1 and MAT1–2 in a collection of 311 isolates of *A. fabae* from South Australia. This result may be due to the sample size and the origin of the isolates that was mainly from field trials rather than commercial crops.

Morphological characterization of 122 isolates among the whole population revealed a high level of diversity. These results support those of Kharrat et al. (2000) who reported morpho-biological diversity after evaluating the aggressiveness and some morphological traits of an international collection of *A. fabae* isolates. This diversity was also reported by Maurin (1989) on conidia originating from the same pycnidia. Kharrat et al. (2000) and Maurin (1989) did not establish any link between the studied parameters. Contrarily, our study was able to establish correlations between the different parameters through a PCA. The correlation established between the different phenotypic parameters through PCA concluded with three groups (MG1, MG2 and MG3) according to their morphological parameters: the frequency of one-celled and two celled conidia and the dimensions of the different conidia types (length and width). Similarly, pathogenic variability was revealed between 122 isolates on the faba bean cv. Badii. This variability was observed for incubation period (IP), and for the disease scores (S1, S4, and S9). Our results confirmed those obtained in previous studies (Hanounik and Robertson, 1989; Maurin, 1989; Rashid et al., 1991; Kharrat et al., 2000; Bessaidi et al., 2017). Maurin (1989) assessed pathogenic variability as well as sculptural criteria (morphology, growth, fruiting *in vitro*). However, he was not able to define pathotypes given the diversity of characters and the instability of the strains since the colonies presented variant sectors, possibly due to the progeny of single spore isolates being heterogeneous. Hanounik and Robertson (1989) showed consistently significant differential interactions among faba bean lines tested against eight isolates of *A. fabae* from Syria. A similar result was obtained by Rashid et al. (1991) when they tested 19 inbred lines against 5 isolates of *A. fabae.* Kharrat et al. (2000) scored six lines for their resistance to three isolates of *A. fabae* on both stems and leaves and they concluded that there was a differential interaction between lines and isolates and also a differential interaction between organ (stem or leaves) and isolate. None of these studies used the PCA tool to group the isolates into aggressiveness groups. This study started initially with 144 isolates but only 122 isolates gave usable results. However according to Pallant (2011) ideally, there should be at least 150 cases for PCA and there should be a ratio of at least five cases for each variable which is not the case for morphological groups in this study. Hence the morphological traits of MG3 may not be a good indicator of aggressiveness or mating type and a study on a larger population may provide a clearer response. MFCA analysis distinguished five different groups based on morphological traits, mating type and aggressiveness level. The most interesting result is that all the highly aggressive isolates were classified in one group: G5 which is composed of MAT1-2 isolates. Some of these have the same frequency of one or two-celled conidia, medium dimensions and relatively long one-celled conidia (MG2) while others in G5 have a high frequency of two-celled conidia, large and long conidia (MG1). Most isolates with a high frequency of one-celled conidia were grouped in G3 and linked to MAT1-2 mating type and were moderately pathogenic (AG2). Isolates from G2 and G4 have the same aggressiveness level (moderately aggressive: AG2) but have different morphological traits. Such results suggest that there is no specific traits linked to aggressiveness since the most aggressive isolates had diverse and the most frequent morphological traits (MG1 and MG2). But it seems that aggressiveness is linked to MAT1-2. Where MAT1-2 is predominant, the spatial and temporal dynamics of the epidemic is rapid and the faba bean crop is exposed to a high disease risk because of the high aggressiveness of this mating type. Rigorous monitoring of the fields is needed in such cases to overcome the epidemic. As supposed by Omri Ben Youssef et al. (2019) our results are an additional argument to support the hypothesis that MAT1-2 fitness seems to be higher than that of MAT1-1, particularly in Bizerte, Jendouba and Kef regions. This hypothesis can be confirmed by further studies to compare the biology of MAT1-1 and MAT1-2 that can identify how climatic conditions and host affect both *in vitro* mating types, mycelium growth, sporulation and germination, and *in vivo* necrosis development, pycnidium genesis, host infection and spatial dispersion. The high aggressiveness linked to MAT1-2 revealed in this study suggests a higher risk for faba bean if contaminated with MAT1-2 than with MAT1-1. As seeds constitute one of the main inoculum sources (Tivoli and Banniza, 2007), seed movement could be a risk of long-distance dispersion by transferring highly pathogenic MAT1-2 isolates from one region to another. Limiting seed transfer and seed health management could avoid such risk. Furthermore, faba bean breeding program should orientate disease resistance selection mainly toward MAT1-2 to be effective at the on-farm level.

## Data availability statement

The original contributions presented in the study are included in the article/**Supplementary Material**. Further inquiries can be directed to the corresponding author.

## Author contributions

ZB, IH, MK, and AM generated the experimental data. NO designed supervised the experiments, and performed statistical analysis and drafted the manuscript. KM and CLM: coordinated and supervised the manuscript redaction. All authors contributed to the article and approved the submitted version.
